# Treatment of Morganella morganii-Associated Non-healing Diabetic Foot Ulcer With Vaporous Hyperoxia Therapy: A Case Report

**DOI:** 10.7759/cureus.60413

**Published:** 2024-05-16

**Authors:** Afrah S Abedi, Jacob L McElroy, Vladimir Valencia, Rachel M Worcester, Zhi J Yu

**Affiliations:** 1 School of Medicine, Lake Erie College of Osteopathic Medicine, Bradenton, USA; 2 Family Medicine, Ascension St. Vincent's Riverside Hospital, Jacksonville, USA

**Keywords:** diabetes mellitus, vaporox, vaporous hyperoxia therapy, morganella morganii, diabetes foot ulcer

## Abstract

Diabetic foot ulcers represent a significant complication of diabetes mellitus, characterized by mechanical changes of bony architecture often leading to chronic wounds with increased risk of infection and impaired healing. *Morganella morganii*, a Gram-negative bacterium, is one of the pathogens found in infected diabetic foot ulcers. It is a human gastrointestinal commensal organism that may cause widespread deadly infections. This report discusses the case of a 76-year-old male with diabetes mellitus who presented with *M. morganii* diabetic foot ulcer to an in-patient rehabilitation facility. Despite conventional wound care and antibiotic therapy, the ulcer failed to improve. The management approach for this patient consisted of a rehabilitation modality called Vaporox, a machine that utilizes vaporous hyperoxia therapy (VHT), as it combines ultrasonic mist and high concentration of oxygen to fasten revascularization and healing. This case highlights the potential efficacy of VHT as an adjunctive therapy for the management of diabetic foot ulcers, particularly those complicated by pathogens, such as *M. morganii*.

## Introduction

Diabetic foot ulcers are a common manifestation of diabetes mellitus, with the pathogenesis of its formation being a multifactorial process. The persistent hyperglycemic state promotes the development of atherosclerotic plaques and endothelial cell dysfunction [1]. Atherosclerotic plaque development leads to diminished blood flow and compromises distal blood supply to the lower extremities. Ensuing ischemia leads to the formation of an ulcer and rendering of the ulcer susceptible to infection [2]. Neuropathy is another important contributor to the pathogenesis of diabetic foot ulcers as inappropriate glucose levels diminish the activity of enzymes responsible for neuronal insulation and conduction. Insufficient proprioceptive input from the lower extremities leads to altered body mechanics and promotes the formation of a callus. This callus, in association with the vicious side effects of peripheral artery disease, contributes to the development of diabetic foot ulcers [1,2]. As the incidence of diabetes mellitus is rapidly increasing, complications are also on the rise with diabetic foot ulcers causing significant morbidity and mortality. Studies have shown that diabetic foot ulcers are often polymicrobial, but *Staphylococcus aureus* continues to be the main causative pathogen [3]. However, the predominance of *Enterobacteriaceae* family has been reported as the largest group of Gram-negative bacteria causing diabetic foot infections, with *Morganella morganii* being one of the most common of this group [4,5,6]. 

*M. morganii* is a facultative anaerobic, Gram-negative rod that is a natural inhabitant of the gastrointestinal tract and commonly found in the environment [6]. It commonly manifests as urinary tract infections and pyogenic skin infections; however, it is also deemed responsible for infected diabetic foot ulcers. This opportunistic pathogen may cause a variety of infections, such as bacteremia, sepsis, abscess, and cellulitis, that can severely affect the immunocompromised [7,8]. Therefore, the mortality of *M. morganii* bloodstream infection remains 38.3-42% in reported cases, requiring prompt treatment [3,7,8]. An innovative therapy that has begun to gain recognition in the management of diabetic foot ulcers is vaporous hyperoxia therapy (VHT).

Vaporox, a VHT machine, is a medical device system to heal chronic wounds, such as diabetic foot ulcers. It is a low-frequency, non-contact, non-thermal ultrasound treatment [9]. Vaporox creates an isolated chamber that delivers hydrating vapor and concentrated oxygen. This oxygen chamber increases blood flow to the wound, increasing the delivery of cells and nutrients responsible for wound repair. This accelerates the production of collagen at the wound, providing the framework for closure [10]. We present a case of a chronic non-healing *M. morganii* plantar foot ulcer in a patient with longstanding diabetes mellitus that was managed by VHT, a safe and effective advanced wound care technology.

## Case presentation

This patient is a 76-year-old male who presented at an inpatient rehabilitation facility following a cervical discectomy and fusion for significant progressive cervical myelopathy with bilateral upper and lower extremity weakness, paresthesias, pain, and myopathy. At the facility, he presented with severe hyperglycemia and was administered Lantus and short-acting insulin without much improvement. Despite medical intervention, his blood sugar was noted to be 600 mg/dL and subsequently transported to the emergency room. During admission, the patient's lab values exhibited negative islet cells and glutamic acid decarboxylase antibody (GADAb) with C-peptide <0.1 ng/mL with glucose 175 mg/dL.

The patient has had type 2 diabetes mellitus for the past 45 years with past episodes of diabetic ketoacidosis. He has an established medical history of severe malnutrition, chronic congestive heart failure with diastolic dysfunction, atrial fibrillation, polyneuropathy, and coronary artery disease. The patient has been in and out of long-term nursing homes and rehabilitation centers medicating with subcutaneous insulin. His current regimen includes Lantus 11 units twice daily and NovoLog eight units with meals and a low-dose sliding scale. His blood sugar levels trends vary, spiking up to 400 mg/dL and going as low as 70 mg/dL.

**Figure 1:**
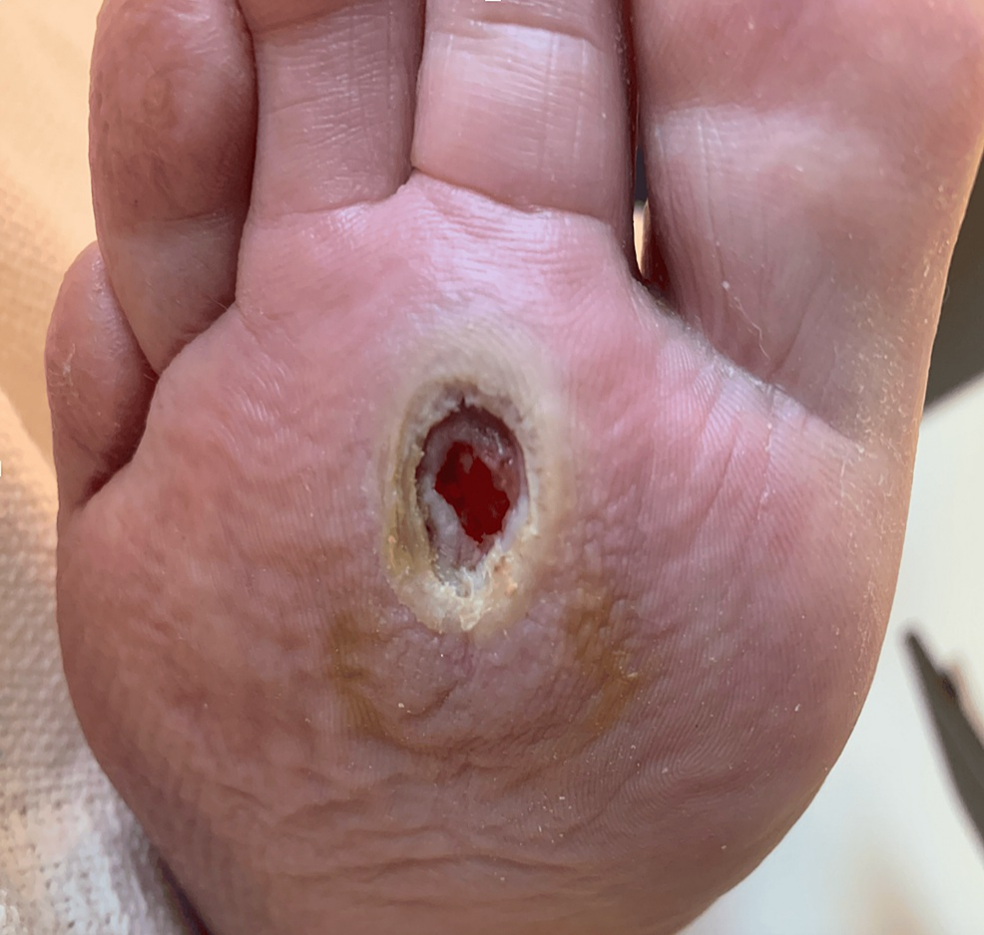
Chronic non-healing plantar ulcer before treatment

The patient was admitted to physical therapy, and the main issue from a clinical standpoint expressed as diabetic noncompliance and dyscontrol, leading to a consult from endocrinology. Although his diabetes mellitus began to be controlled, he eventually developed a blister on the bottom of his right plantar foot, which perpetually worsened. The insidious onset of the foot ulcer began once it popped, as shown in Figure 1.

Wound care began following the patient. The right plantar foot ulcer was cultured with a Gram stain and yielded heavy growth of normal skin flora (Gram-positive rods and Gram-positive cocci) and Gram-negative rods, as shown in Table 1. *M. morganii* was the sole isolate found in the heavy growth of Gram-negative rods. Sensitivity analysis was performed, as reported in Table 2. The isolate, *M. morganii*, displayed the highest sensitivity against trimethoprim-sulfamethoxazole (TMP/SMX). The patient was started on oral TMP/SMX instead of intravenous antibiotics as per the wound care team. Extensive debridement was done on the initial visit. Although TMP/SMX was reported with the highest sensitivity against *M. morganii*, the patient's diabetic foot ulcer did not have any significant improvement despite antibiotic treatment. 

**Table 1:** Positive culture of Morganella morganii on the right plantar foot ulcer

Site	Gram stain	Result	Isolate
Right foot	3+ Gram-negative rods	Heavy growth of normal skin flora	*M. morganii* ssp. *sibonii* (Isolate 1)
	2+ Gram-positive cocci	Heavy growth of Gram-negative rods	
	1+ Gram-positive rods		
	1+ WBCs		

**Table 2:** Sensitivity Analysis for Morganella morganii (Isolate 1) MIC: minimum inhibitory concentration, S: sensitive, I: intermediate, R: resistant

Antibiotic	MIC	Interpretation
Ampicillin	>=32	R
Ampicillin/sulbactam	>=32	R
Cefazolin	>=64	R
Cefepime	4	S
Ceftazidime	16	I
Ceftriaxone	>=64	R
Ciprofloxacin	>=4	R
Ertapenem	<=0.5	S
Gentamicin	<=1	S
Levofloxacin	>=8	R
Piperacillin/tazobactam	<=4	S
Trimethoprim/sulfamethoxazole	<=20	S

**Figure 2:**
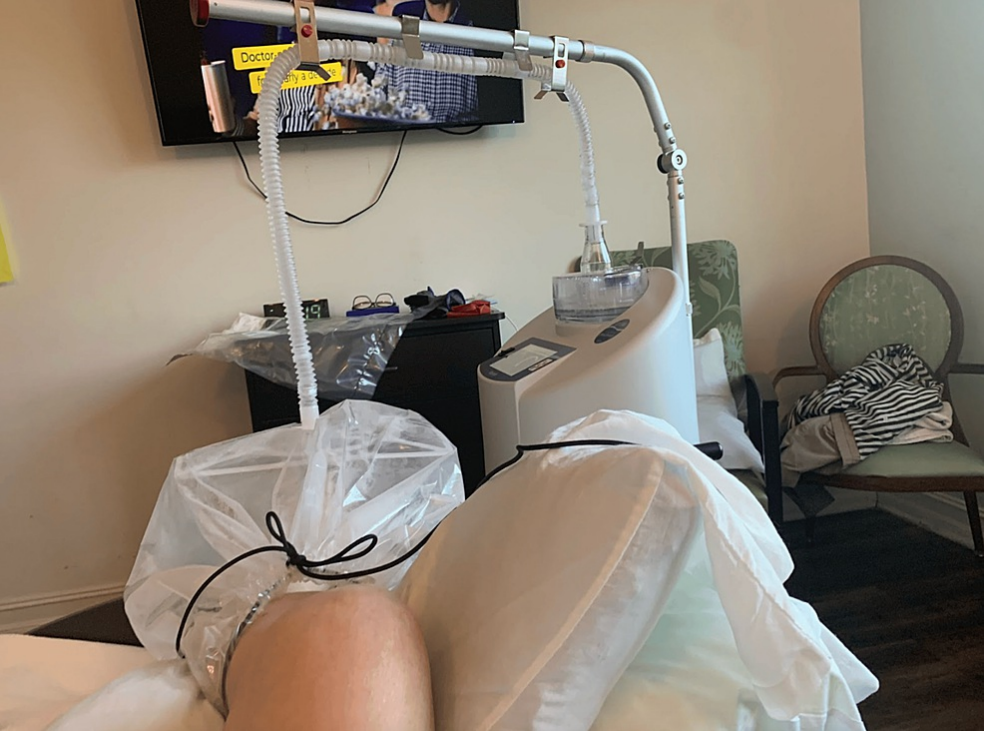
Vaporox treatment setup

The patient reported generalized weakness but denied any pain, fevers, or chills. At the inpatient rehabilitation center, the wound care team assessed the diabetic foot ulcer and decided to utilize a different therapeutic approach as antibiotics were not able to heal the ulcer. They decided to treat the wound using Vaporox to deliver VHT. Vaporox treatment was arranged and VHT was promptly administered, as shown in Figure 2.

**Figure 3:**
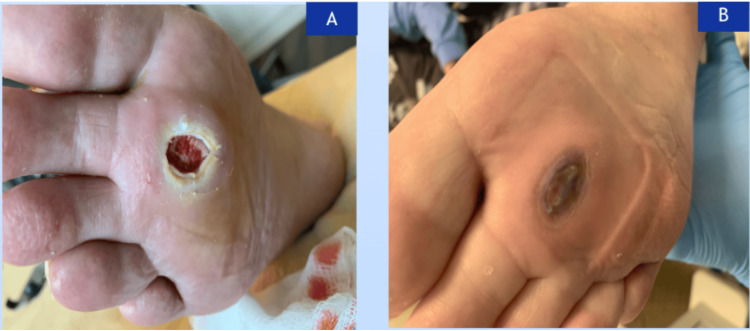
A) First treatment with Vaprox on 11/28/23. B) Last treatment with Vaporox on 2/21/24.

Vaporous hyperoxia wound care treatment was provided in 10-minute cycles of ultrasonic sterile water mist and five minutes of concentrated oxygen. The patient was positioned seated with the right lower extremity elevated. The post-treatment consisted of wiping the wound bed dry, applying hydrofera dressing on the wound, and covering it with 4 x 4 sterile dressing. This treatment was administered twice every week. Diabetic foot ulcer was documented by capturing images when improvement was prominent, as Figure 3A depicts the plantar foot ulcer after the first session of VHT and Figure 3B depicts a healed wound ulcer after the last session of VHT as declared by the patient's wound care team.

Ambulation was not compromised during the treatment course. The patient was provided with daily wound dressings to provide a barrier for infection and prevent injuries. Once VHT was complete, the patient was advised to continue proper wound care and monitor blood sugar levels.

## Discussion

A remarkable 37.3 million people in the United States have diabetes while 18.6 million people are affected by a diabetic foot ulcer [1,11]. Several factors, such as neurological, vascular, and biomechanical, contribute to ulceration [11]. On average, 50-60% of ulcers lead to infection and 20% of moderate to severe infections can require amputations. These ulcers precede 80% of lower extremity amputations among people diagnosed with diabetes and are associated with an increased risk of death. Diabetic foot ulcers have become a debilitating manifestation for diabetic patients and pose a significant burden on the healthcare system as these ulcers precede 80% of lower extremity amputations and are associated with an increased risk of death. Current standard simple wound care for diabetic foot ulcers has culminated in a holistic treatment approach [1]. The main components of simple wound care revolve around the importance of strict glycemic control and routine dressing changes. However, surgical debridement, alleviating ulcer pressure, and prioritizing treatment for lower extremity ischemia and infection are first-line therapies [10]. Patients are advised to have daily cleanings and are strongly discouraged from adding pressure at the ulcer site. The necessity to have daily treatment puts a burden on both healthcare resources and personnel. A retrospective cohort study of patients with diabetic foot ulcers found the average healing time to be 113 days [1]. This calls attention to the importance of innovative treatment methods that will shorten the duration for sufficient wound closure.

Vaporox is a portable, therapeutic machine that delivers VHT to chronic wounds [9]. VHT has revolutionized the management of chronic wounds and given patients a sense of newfound hope. The development of VHT is rooted in the physiologic mechanisms responsible for wound repair. Oxygen plays an essential role in this process that aids in the delivery of cells and nutrients necessary for angiogenesis at the wound site. The angiogenesis is in part regulated by transcription factors known as hypoxia-inducible factors (HIFs) [12]. This transcription factor is responsible for activating the genes that are required for wound repair. HIF-1 also induces the release of cytokines that contribute to the reparative process by further initiating the mobilization of bone marrow-derived angiogenic cells [13].

Furthermore, VHT has been used for chronic wounds ranging from diabetic foot ulcers, pressure ulcers, venous insufficiency ulcers, and burns. Clinical trials have shown that a staggering 84% of chronic wounds healed within a 20-week period, compared to just 31% of patients who underwent standard wound care therapy [10]. The rate of healing with VHT combined with standard wound care drastically increases the rate of healing of diabetic foot ulcers, specifically 2.85 times more compared to standard wound care alone. These trials demonstrate the profound impact VHT may play in the role of chronic wound therapy going forward. Another study exploring the use of oxygen therapy and its impact in expediting the healing process for diabetic foot ulcers was conducted using transdermal continuous oxygen therapy [14]. This study randomly selected patients with diabetic foot ulcers to be treated with continuous administration of 98% oxygen at the wound using a 15-day device. Patients were evaluated for complete wound closure at week 12. It was found that 56% of patients receiving oxygen therapy reached full wound closure compared to just 49% of patients in the control group. This study further validates the rationale behind oxygen therapy and its utilization in wound management.VHT has become a proven therapy that expedites the process of adequate wound closure [9]. As VHT targets the delivery of oxygen-rich vapors to an affected area, it has become revolutionary in the treatment of diabetic foot ulcers prompting water wound healing and reducing the risk of complications, especially with pathogens, such as *M. morganii*.

Although a human commensal organism, *M. morganii* is becoming increasingly recognized as a public health threat [7]. In a study conducted by Ghosh et al., the researchers analyzed a case of a diabetic heel ulcer that did not respond to antibiotic therapy, which caused the infection to progress to involve the entire leg [15]. Imaging depicted worsening deep infection into the fascial planes, specifically showing the presence of gas gangrene, and the blood cultures yielded *M. morganii*. The diabetic foot ulcer infection developed in such a rapid manner that it led to the demise of the patient within 72 hours of presentation. In addition, Cetin et al. highlighted a case in which a diabetic foot ulcer developed septic arthritis in the left knee [16]. The patient, with an existing diabetic foot ulcer, presented with fevers, malaise, and severe pain in his left knee with no concurrent history of trauma. Arthrocentesis was performed on his knee and bacterial identification of the synovial fluid indicated *M. morganii*, yielding the same results from the ulcer. Hematogenous spread occurred from the diabetic foot ulcer to the knee, indicating the transformation of soft tissue infection. Furthermore, Alsaadi et. al. reported from various clinical cases that *M. morganii* causes invasive infections in humans at various sites including tissues [7]. *M. morganii* infections can occur in patients of all ages, from neonates to the elderly. Treatment includes antibiotic therapy, debridement, and drainage. Although this management can heal *M. morganii* infections, several cases reported that antibiotics and aggressive surgical debridement did not decrease the mortality rate of these infections. Alsaadi et al. conducted a retrospective cohort study with positive *M. morganii *blood cultures. *M. morganii* bacteremia was identified with comorbidities, such as diabetes, hypertension, and kidney disease. The outcome of this study presented that 41% of the cases were reported as in-hospital mortality while 32% did not survive past 30 days since the development of *M. morganii* bloodstream infections. *M. morganii* can develop into a serious infection from existing comorbidities, but more epidemiology research is needed to fully understand its virulence capabilities.

In the case we present, the patient began VHT once *M. morganii* did not respond to antibiotic therapy that was deemed necessary in the antibiotic sensitivity analysis. Although the VHT cannot yield immediate results, it does allow for the healing of the diabetic foot ulcer to prevent the occurrence of widespread infections in the body that *M. morganii* can cause. In the setting of severe infection, such as the *M. morganii*-associated diabetic foot ulcer, VHT has proven that it is capable of containing infection in patients unresponsive to standard wound care alone.

## Conclusions

VHT emerges as a promising adjunctive treatment for diabetic foot ulcers associated with *M. morganii* infection. Through its targeted delivery of oxygen, this innovative therapy creates an inhospitable environment for the growth and survival of *M. morganii*, an opportunistic pathogen that can lead to widespread debilitating infections. VHT not only addresses the immediate concerns of infection but also promotes the long-term management of diabetic foot ulcers. Nonetheless, the potential of VHT to revolutionize the treatment regimen for diabetic foot ulcers underscores its significance in improving patient outcomes and reducing the burden of complications.
